# Calculation of exchange integrals and Curie temperature for La-substituted barium hexaferrites

**DOI:** 10.1038/srep36200

**Published:** 2016-10-31

**Authors:** Chuanjian Wu, Zhong Yu, Ke Sun, Jinlan Nie, Rongdi Guo, Hai Liu, Xiaona Jiang, Zhongwen Lan

**Affiliations:** 1 State Key Laboratory of Electronic Thin Films and Integrated Devices, University of Electronic Science and Technology of China, Chengdu 610054, China; 2 Department of Applied Physics, University of Electronic Science and Technology of China, Chengdu 610054, China

## Abstract

As the macro behavior of the strength of exchange interaction, state of the art of Curie temperature *T*_c_, which is directly proportional to the exchange integrals, makes sense to the high-frequency and high-reliability microwave devices. Challenge remains as finding a quantitative way to reveal the relationship between the Curie temperature and the exchange integrals for doped barium hexaferrites. Here in this report, for La-substituted barium hexaferrites, the electronic structure has been determined by the density functional theory (DFT) and generalized gradient approximation (GGA). By means of the comparison between the ground and relative state, thirteen exchange integrals have been calculated as a function of the effective value *U*_eff_. Furthermore, based on the Heisenberg model, the molecular field approximation (MFA) and random phase approximation (RPA), which provide an upper and lower bound of the Curie temperature *T*_c_, have been adopted to deduce the Curie temperature *T*_c_. In addition, the Curie temperature *T*_c_ derived from the MFA are coincided well with the experimental data. Finally, the strength of superexchange interaction mainly depends on 2*b*-4*f*_1_, 4*f*_2_-12*k*, 2*a*-4*f*_1_, and 4*f*_1_-12*k* interactions.

Owing to the large magnetocrystalline anisotropy, high Curie temperature *T*_c_ and saturation magnetization *M*_s_, barium hexaferrites [BaFe_12_O_19_, BaM] are of great interest for magnetic recording, microwave magnetic devices, and permanent magnets^1^,^2^,^3^. Many schemes^3^,^4^,^5^,^6^,^7^ have been attempted in the past few decades to improve the intrinsic magnetic properties of barium hexaferrites. The typical representative is La-based substitutions^8^,^9^,^10^. La-substitutions, which are benefit to improve the saturation magnetization and magnetocrystalline anisotropy, however, are detrimental to enhance the Curie temperature^8^,^9^,^10^,^11^. With the requirements of wide operating temperature range of microwave devices and components, it is very important to quantitatively explore the relationship between the Curie temperature *T*_c_ and exchange integrals.

The Heisenberg model provides an opportunity to realize account for a large amount of the basic physical laws of ferrites from a phenomenological description. Especially, for a wide range of spinel ferrites^12^,^13^, the exchange interactions have been investigated based on the molecular field theory employing the generalized gradient approximation (GGA) and local spin density approximation (LSDA) methods. So far, the work of the relationship between the Curie temperature *T*_c_ and exchange integrals for barium hexaferrites has been mainly concentrated on the undoped samples using the nonlinear fitting methods: Isalgué *et al*.^14^ suggested that 12*k* sublattice of barium hexaferrites is subject to a strong exchange interaction for the sake of the link between R (R*) and S (S*) blocks, and Grill *et al*.^15^ confirmed that Fe^3+^_2*b*_-O-Fe^3+^_4*f*2_, Fe^3+^_2*a*_-O-Fe^3+^_4*f*1_, Fe^3+^_4*f*2_-O-Fe^3+^_12*k*_, and Fe^3+^_4*f*1_-O-Fe^3+^_12*k*_ triads demonstrate the comparatively strong exchange coupling. Unfortunately, the above calculations neglect the intra-sublattice interactions and strongly correlated 3*d* electrons of Fe, and then overestimate the exchange integrals and Curie temperature *T*_c_.

In short, in terms of La-substituted barium hexaferrites, there are seldom researches that quantitatively explore the relationship between the Curie temperature *T*_c_ and exchange integrals. And the exchange integrals as a function of the effective value *U*_eff_ have not been also investigated completely within the framework of the density functional theory. Furthermore, the Curie temperature *T*_c_ has not been realized based on the exchange integrals by the molecular field approximation (MFA) and random phase approximation (RPA) methods. So this paper would focus on solving these issues.

## Results and Discussion

### Crystal and Magnetic structure

**Figure 1:**
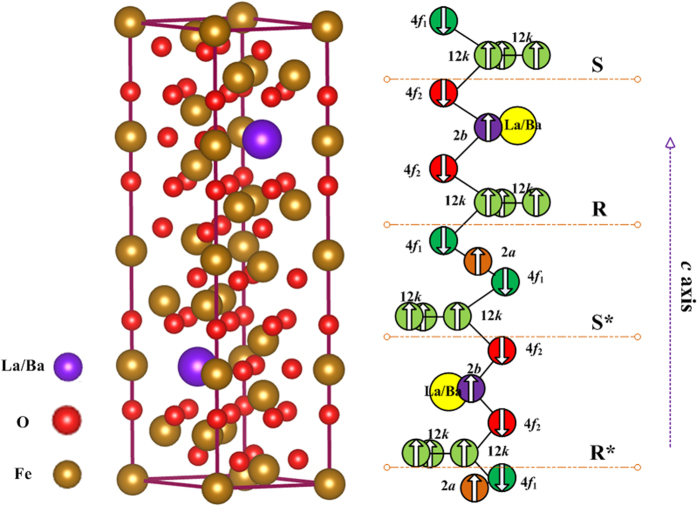
The unit cell and spin configurations for Ba_1−*x*_La_*x*_Fe_12_O_19_: Purple, red, and gold spheres denote Ba/La, O and Fe atoms. The arrows represent the local magnetic moment at each atom site.

**Figure 2:**
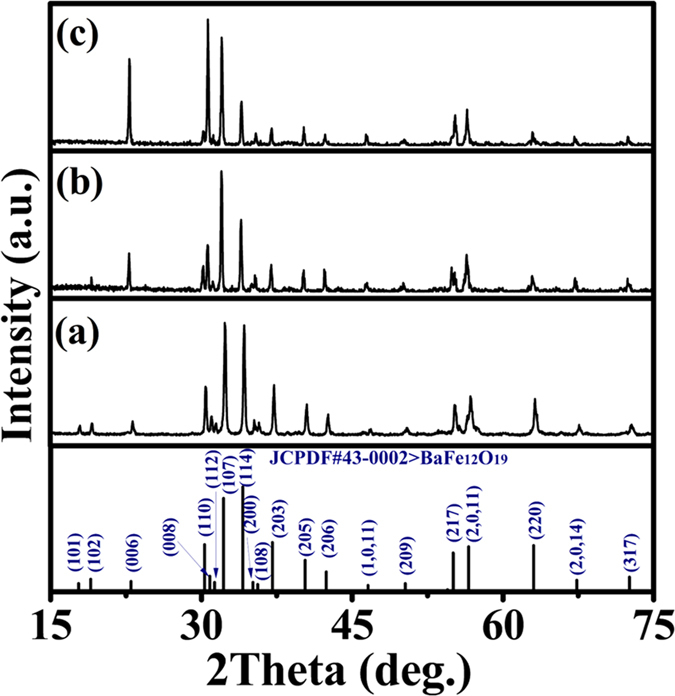
The X-ray diffraction patterns for Ba_1−*x*_La_*x*_Fe_12_O_19_ samples. (**a**) *x* = 0.0, (**b**) *x* = 0.5, (**c**) *x* = 1.0. And the main (*hkl*) peaks from JCPDF Card No. 43–0002 for BaFe_12_O_19_ are also plotted.

**Figure 3:**
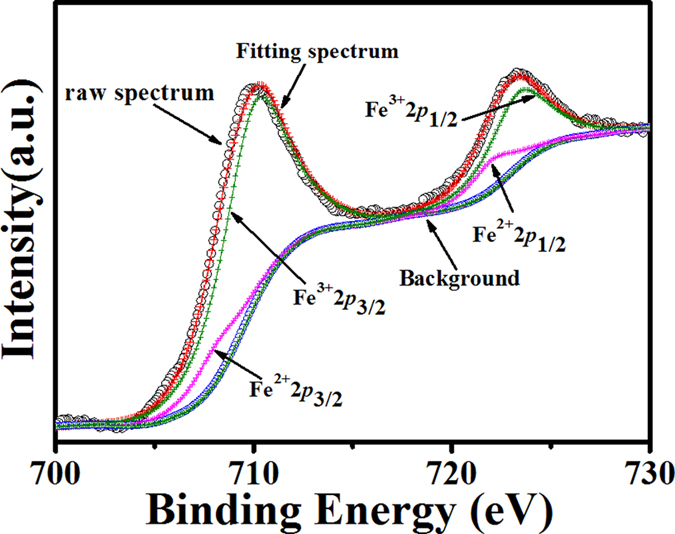
The representative XPS spectra of iron ions for LaFe_12_O_19_ sample.

The crystal structure of M-type hexaferrites could be described as SRS*R*, where *S* = (Fe_6_^3+^O^2−^_8_)^2+^ is a spinel block with only two layers, and *R* = (Ba^2+^Fe_6_^3+^O^2−^_11_)^2−^ is a barium containing hexagonal block with three oxygen layers: S* and R* are obtained from *S* and *R* blocks, respectively, by a rotation of 180° about *c* axis^16^. As shown in Fig. 1, 24 Fe^3+^ ions of M-type hexaferrites are distributed in five different sublattices: 3 parallel sites (12*k*, 2*a* and 2*b*) and 2 antiparallel sites (4*f*_1_ and 4*f*_2_)^17^. La-based substitutions could contribute to some Fe^3+^ transforming into Fe^2+^ at the 2*a* and 4*f*_2_ sites^18^. The X-ray diffraction (XRD) patterns indicated that the compounds are crystallized in a magnetoplumbite hexagonal structure (see Fig. 2). According to the relationship between the cation distribution and the magnetic moments at 0 K^19^,^20^, the cation distribution of La-substituted barium hexaferrites is summarized in Table 1. In order to conform the results, the photoelectron counting area of Fe^2+^ and Fe^3+^ ions is presented in Fig. 3, and the molar ratio of Fe^2+^/(Fe^2+^+Fe^3+^) for LaFe_12_O_19_ sample is approximately 9%.

### Ab initio calculation of exchange interactions

For the exchange interaction between two spins in the isotropic Heisenberg mode, the ferrimagnetic spin configurations (up or down) are considered. The exchange energy per unit cell in the complex system with *N* magnetic sublattices could be then written as


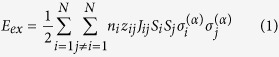


where *n*_i_ is the number of *i*th sublattice, *z*_ij_ is the number of neighboring sites in *j*th sublattice to *i*th sublattice, *J*_ij_ is the exchange integrals, *S*_i_ and *S*_j_ represent the spins in the *i*th and *j*th sublattices, 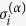
and 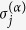
are equal to ±1, and the index *α* is the spin arrangement of the sublattices. We denote *α*_0_ as the ground state, and *α*_n_ (n ≠ 0) as the relative state. The neighboring *z*_ij_ and corresponding distance *r*_ij_ for five sublattices are given in Table 2. The difference between the exchange energy of *α*_n_ and *α*_0_ is


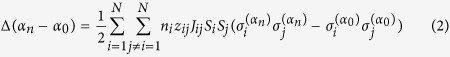


Note that *n*_*i*_*z*_*ij*_ = *n*_*j*_*z*_*ji*_. When the spin of a single sublattice is inverted relative to the ground state, we get


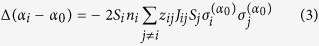


When the spins of two sublattices inverted relative to the ground state are considered, we then obtain.


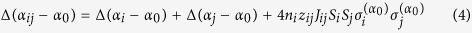


Thereby the exchange integral could be given by


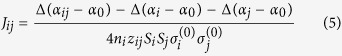


**Figure 4:**
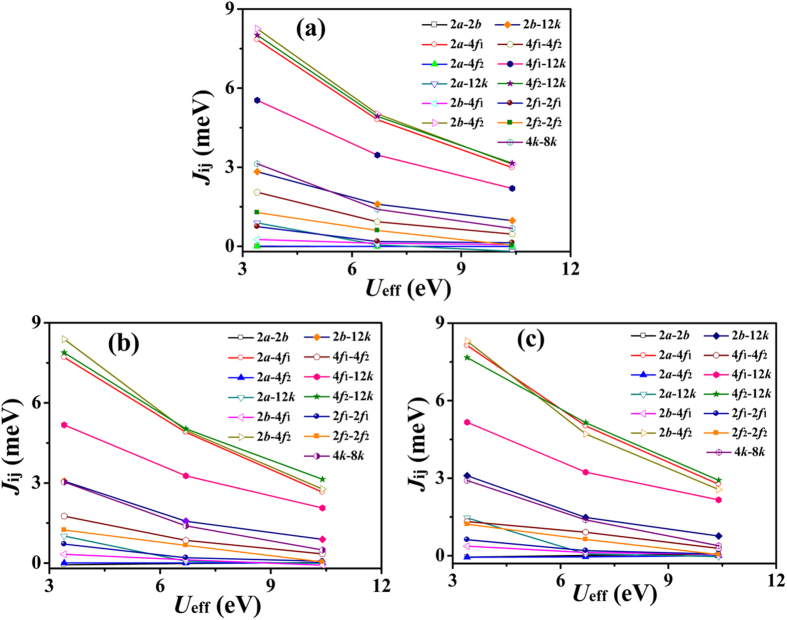
The exchange integrals as a function of *U*_eff_ for Ba_1−*x*_La_*x*_Fe_12_O_19_ samples. (**a**) *x* = 0.0, (**b**) *x* = 0.5, (**c**) *x* = 1.0.

As mentioned above, the Fe^3+^ and Fe^2+^ ions in the five sublattices are anti-ferromagnetically coupled with each other. The orbit angular momentum is frozen and hence the magnetism of the Fe^3+^ and Fe^2+^ ions mainly results from the spin angular momentum *S* = 2.5 and *S* = 2.0, respectively^20^. For the mixed valence Ba/La hexaferrites, the spin angular momentum *S*_*i*_ in the *i*th sublattice could be assumed to be.


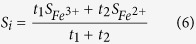


where *t*_1_ and *t*_2_ are the Fe^3+^ and Fe^2+^ numbers in the *i*th sublattice, respectively. Since the exchange interactions between two magnetic ions decrease with the increase of the corresponding distance, the distance more than 4 Å could be neglected^21^. As shown in Table 2, the distance of neighboring 4*f*_1_, 4*f*_2_, and 12*k* sublattices is smaller than 4 Å, and in fact these sublattices have also the nearest Fe neighbors in the same sublattices. Ten inter-sublattice interactions (2*a*-2*b*, 2*a*-4*f*_1_, 2*a*-4*f*_2_, 2*a*-12*k*, 2*b*-4*f*_1_, 2*b*-4*f*_2_, 2*b*-12*k*, 4*f*_1_-4*f*_2_, 4*f*_1_-12*k*, and 4*f*_2_-12*k*) and three intra-sublattice interactions (4*f*_1_-4*f*_1_, 4*f*_2_-4*f*_2_, and 12*k*-12*k*) were thus considered. For intra-sublattice interaction calculations, in order to preserve the highest symmetry, one divided the 4*f*_1_, 4*f*_2_, and 12*k* sublattices into (2*f*_1_, 2*f*_1_), (2*f*_2_, 2*f*_2_), and (4*k*, 8*k*), respectively. The representative calculations (*x* = 0.5) of energy difference per unit cell for each relative state are given in Table 3. According to the Eq. (5), thirteen exchange integrals as a function of *U*_eff_ (see Fig. 4) could be obtained. The 2*a*-2*b* and 2*a*-4*f*_2_ interactions are very small with |*J*_2*a*-2*b*_| and |*J*_2*a*-4*f*2_| < 0.05, which is ascribed to the Fe_2*a*_-O-Fe_2*b*_, _*a*_nd Fe_2*a*_-O-Fe_4*f*2 *a*_ngles approach 90°^14^. The different ionic radii of La^3+^ (1.22 Å), and Ba^2+^ (1.47 Å)^11^ causes some changes in exchange interactions of Ba/La hexaferrites: The 2*a*-4*f*_1_, 2*a*-12*k*, 2*b*-4*f*_1_, and 2*b*-_1_2*k* interactions increase, and whilst the 4*f*_1_-4*f*_2_, 4*f*_1_-_1_2*k*, 4*f*_2_-_1_2*k*, 2*f*_1_-_2_*f*_1_, 2*f*_2_-2*f*_2_, and 4*k*-8*k* interactions decrease with the increase of La concentrations. It is emphasized, however, 2*b*-4*f*_2_ interaction has a slight change (about 0.1 eV). This is associated with the strong effects of barium or lanthanum ions on the nearest neighboring iron ions (2*b* and 4*f*_2_ su*b*lattices)^22^.

### Calculations of Curie temperature

In the following one calculated the Curie temperature *T*_c_ employing the Heisenberg Hamiltonian. The common calculations of Curie temperature *T*_c_ derived from the Heisenberg model contain the mean-filed approximation (MFA) and random-phase approximation (RPA) methods^23^.

The mean-field approximation is based on the notion of single-spin excitations, and the Hamiltonian is^24^


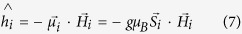


where *g* and *μ*_B_ are the Lande factor and Bohr magneton, respectively. The molecular field 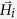 could be thus defined as


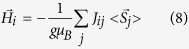


with


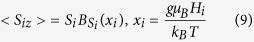


where 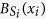 is the Brillouin function, *k*_*B*_ is the Boltzmann constant, and *T* is the temperature in K. When *T* is very high, such as *k*_*B*_*T*  ≫ *μ*_B_*H*_i_, the 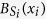 and <*S*_*iz*_> are rewritten as


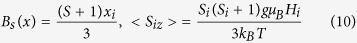


Introducing the exchange integral *J*_ij_, we get


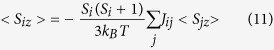


i.e.,


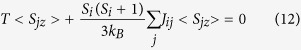


which has nonzero solution only if the determinant


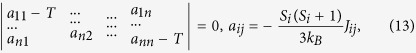


In fifth order systems, there are five solutions in Eq. (13). The highest positive *T* is the desired Curie temperature *T*_c_.

In the random-phase approximation, it is assumed that the thermal disordering is achieved by the excitation of independent spin waves^25^. The equation of motion for the Green function (analogically to Callen)^26^ is given that


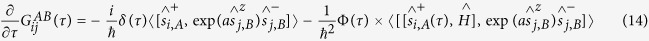


where *α, δ*(τ), Φ(τ) and 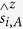 are an auxiliary, unit-impulse function, unit-step function, and spin operators operating in the unit cell *i* at the basis site. Here 
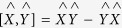
 is a commutator, 
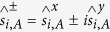
, and the mean value 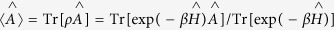 with 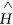 being the Heisenberg Hamiltonian and *β* = 1/(*k*_B_*T*). According to Tyablikov 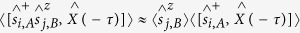
27, the double commutator could be simplified by applying the RPA decoupling. For convenience, the matrix *N*(*q*) could be defined as


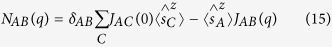


After performing time and lattice Fourier transformations, the Green’s function could be expressed by the following form


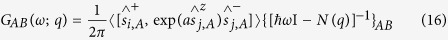


where I is unity matrix. Based on the solutions of Green’s function, as described in Ref. 28, the self-consistent equation for the mean values could be written as


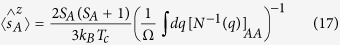


where *T*_c_ is the Curie temperature, *k*_*B*_ is the Boltzmann constant, and *S*_*A*_ is the spin quantum numbers. By the iterative methods to solve this self-consistent set of 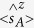, the Curie temperature *T*_c_ could be then obtained.

Table 4 shows the experimental and calculating values of Curie temperature. It is concluded that the experimental Curie temperature is reproduced by calculations for the effective value *U*_eff_ ≈ 6.7 eV, and the MFA and RPA estimations provide an upper and lower bound of the Curie temperature. In the case of La-substituted barium hexaferrites, the *T*_c_ determined by the RPA is in good agreement with the experimental *T*_c_. This could be explicated: the fluctuations of spin wave in MFA (i.e., fluctuations in the magnitudes of the atomic moments) are generally neglected, and hence the arithmetic average takes all the magnon values with the equal weight. While in RPA, this is the harmonic average, and the weight decreases with the increasing spin-wave energy^25^,^28^.

## Conclusions

The composition of Ba_1−*x*_La_*x*_Fe_12_O_19_ (*x* = 0.0, 0.5 and 1.0) were prepared by a conventional ceramic method. Thirteen exchange interactions were calculated by the DFT and GGA + U. With the increase of La contents, the 2*a*-4*f*_1_, 2*a*-12*k*, 2*b*-4*f*_1_, and 2*b*-12*k* interactions increase, the 4*f*_1_-4*f*_2_, 4*f*_1_-12*k*, 4*f*_2_-12*k*, 2*f*_1_-2*f*_1_, 2*f*_2_-2*f*_2_, and 4*k*-8*k* interactions decrease, while the 2*b*-4*f*_2_ interaction has a slight change. The Curie temperature was then calculated using the MFA, and RPA estimations. The RPA is more coincident with the experiments for the effective value *U*_eff_ ≈ 6.7 eV.

## Methods

### Experimental procedures

The compositions of Ba_1−*x*_La_*x*_Fe_12_O_19_ (*x* = 0.0, 0.5 and 1.0) were fabricated by a conventional ceramic method. The analytical-grade raw materials, BaCO_3_, La_2_O_3_, and Fe_2_O_3_ were weighed in stoichiometric proportion and mixed homogeneously in zirconia ball mills for 12 h. The slurries, after being dried, were calcined at 800 °C for 2 h and then second-milled with 3.0 wt% Bi_2_O_3_ for 8 h. After being further dried at 90 °C, the powders were granulated, pressed and sintered at 1050 °C for 2 h in air. Essential for preventing decomposition into Fe_2_O_3_ and LaFeO_3_/BaFe_2_O_4_ is rapid cooling. The X-ray diffraction (XRD) patterns were identified on Bruker D8 Advance X-ray diffractometer with Cu-Kα radiation. The binding energy of iron ions was acquired by X-ray photoelectron spectroscopy (XPS) XSAM800. The hysteresis loops of the samples at 1.8 K (approaching 0K) were measured by Quantum Design SQUID VSM under the applied static magnetic fields up to 6T. The experimental values (E.V.) of Curie temperature for La-substituted barium hexaferrites were measured by the Thermal Gravimetric Analyzer (TGA) under N_2_ atmosphere using a TA-Q50 series analyzer system.

### Computational details

The total energies and forces were calculated using the density-functional theory (DFT) with the Generalized Gradient Approximation (GGA) as parameterized by the Perdew-Burke-Ernzerhof (PBE) in VASP^29^,^30^. In structure optimization, we adopted the Conjugate Gradient (CG) method to optimize the lattice parameters and the position of ions until the force on each ion was less than 0.03 eV/Å. The plane-wave cutoff energy and convergence criteria were 500 eV and 10^−7^ eV, respectively. The reciprocal space was sampled with an 11 × 11 × 1 Monkhorst-Pack mesh^31^. All the calculations were spin polarized according to the Gorter’s ferrimagnetic ordering of the magnetic moments^32^. For improved description of 3*d* electrons in iron ions, the generalized gradient approximation with Coulomb and exchange interaction effects (GGA+U) were employed, where an on-site potential is added to introduce intra-atomic interactions between the strongly correlated electrons^33^. We employed an effective value (*U*_eff_) equal to the difference between the Hubbard parameter *U* and the exchange parameter *J*^34^. To study how the results depend on *U*_eff_, three values (3.4, 6.7, and 10.4 eV) on Fe atoms were adopted on the basis of many rigorous calculations of barium hexaferrites^31^,^35^,^36^.

## Additional Information

**How to cite this article**: Wu, C. *et al*. Calculation of exchange integrals and Curie temperature for La-substituted barium hexaferrites. *Sci. Rep.* **6**, 36200; doi: 10.1038/srep36200 (2016).

**Publisher’s note**: Springer Nature remains neutral with regard to jurisdictional claims in published maps and institutional affiliations.

## Figures and Tables

**Table 1:** The cation distribution for Ba_1−*x*_La_*x*_Fe_12_O_19_ samples.

Samples	*σ*_*s*_ (emu/g)	Cation distribution
*x* = 0.0	100.50	(Ba^2+^Fe_2_^3+^[Fe^3+^]O_3_^2−^ Fe_2_^3+^[(Fe^3+^)(Fe_6_^3+^)]O_16_^2−^)_2_ 24
*x* = 0.5	100.31	(Ba_0.5_^2+^La_0.5_^3+^Fe_1.762_^3+^Fe_0.238_^2+^[Fe^3+^]O_3_^2−^ Fe_2_^3+^[(Fe_0.738_^3+^Fe_0.262_^2+^)(Fe_6_^3+^)]O_16_^2−^)_2_
*x* = 1.0	100.28	(La^3+^Fe_1.508_^3+^Fe_0.492_^2+^[Fe^3+^]O_3_^2−^ Fe_2_^3+^[(Fe_0.492_^3+^Fe_0.508_^2+^)(Fe_6_^3+^)]O_16_^2−^)_2_

**Table 2:** The number of nearest Fe neighbors and corresponding distance for five sublattices in the BaFe_12_O_19_.

	2*a*		2*b*		4*f*_1_		4*f*_2_		12*k*	
	*z*_ij_	*z*_ij_	*z*_ij_	*z*_ij_	*z*_ij_	*r*_ij_	*z*_ij_	*r*_ij_	*z*_ij_	*r*_ij_
2*a*	6	0.589	2	0.580	6	0.346	6	0.557	6	0.305
2*b*	3	0.580	6	0.589	6	0.619	6	0.367	6	0.371
4*f*_1_	2	0.346	3	0.619	3	0.363	1	0.379	6	0.350
									3	0.356
4*f*_2_	3	0.557	3	0.367	1	0.379	1	0.277	6	0.351
12*k*	1	0.305	1	0.371	2	0.350	2	0.351	2	0.291
					1	0.356			2	0.298

**Table 3:** The energy difference △(*U*_eff_) in eV between the ground state and excited state.

*S*_1_	*S*_2_	△(3.4)	△(6.7)	△(10.4)
2*a*	—	0.853	0.607	0.565
2*b*	—	0.802	0.476	0.285
4*f*_1_	—	3.218	1.647	0.951
4*f*_2_	—	3.349	1.814	1.268
12*k*	—	3.986	1.972	1.244
2*a*	2*b*	1.649	1.082	0.853
2*a*	4*f*_1_	1.882	0.861	0.762
2*a*	4*f*_2_	4.201	2.420	1.831
2*a*	12*k*	5.128	2.598	1.800
2*b*	4*f*_1_	3.921	2.086	1.260
2*b*	4*f*_2_	1.694	0.835	0.741
2*b*	12*k*	5.707	2.918	1.795
4*f*_1_	4*f*_2_	6.738	3.545	2.252
4*f*_1_	12*k*	2.548	0.758	0.341
4*f*_2_	12*k*	2.718	0.842	0.673
2*f*_1_	—	1.556	0.809	0.470
2*f*_2_	—	1.645	0.891	0.633
4*k*	—	0.758	0.453	0.267
8*k*	—	2.013	0.963	0.781

**Table 4:** The experimental and calculating Curie temperature as a function of *U*_eff_ for Ba_1−*x*_La_*x*_Fe_12_O_19_ samples.

*U*_eff_ (eV)	*x* = 0.0			*x* = 0.5			*x* = 1.0		
	3.4	6.7	10.4	3.4	6.7	10.4	3.4	6.7	10.4
E.V. (K)	723	723	723	705	705	705	695	695	695
MFA (K)	1514	980	629	1450	951	581	1419	928	579
RPA (K)	1009	653	419	967	634	387	946	619	386
